# Safety and efficacy of four-segmented Rift Valley fever virus in young sheep, goats and cattle

**DOI:** 10.1038/s41541-020-00212-4

**Published:** 2020-07-24

**Authors:** Paul J. Wichgers Schreur, Nadia Oreshkova, Lucien van Keulen, Jet Kant, Sandra van de Water, Pál Soós, Yves Dehon, Anna Kollár, Zoltán Pénzes, Jeroen Kortekaas

**Affiliations:** 1 Department of Virology, Wageningen Bioveterinary Research, Lelystad, The Netherlands; 2 BunyaVax B.V., Lelystad, The Netherlands; 3 Ceva Animal Health, Ceva-Phylaxia, Budapest, Hungary; 4 Laboratory of Virology, Wageningen University and Research, Wageningen, The Netherlands

**Keywords:** Infectious diseases, Vaccines, Virology

## Abstract

Rift Valley fever virus (RVFV) is a mosquito-borne bunyavirus that causes severe and recurrent outbreaks on the African continent and the Arabian Peninsula and continues to expand its habitat. RVFV induces severe disease in newborns and abortion in pregnant ruminants. The viral genome consists of a small (S), medium (M) and large (L) RNA segment of negative polarity. The M segment encodes a glycoprotein precursor protein that is co-translationally cleaved into the two structural glycoproteins Gn and Gc, which are involved in receptor attachment and cell entry. We previously constructed a four-segmented RVFV (RVFV-4s) by splitting the M genome segment into two M-type segments encoding either Gn or Gc. RVFV-4s replicates efficiently in cell culture but was shown to be completely avirulent in mice, lambs and pregnant ewes. Here, we show that a RVFV-4s candidate vaccine for veterinary use (vRVFV-4s) does not disseminate in vaccinated animals, is not shed or spread to the environment and does not revert to virulence. Furthermore, a single vaccination of lambs, goat kids and calves was shown to induce protective immunity against a homologous challenge. Finally, the vaccine was shown to provide full protection against a genetically distinct RVFV strain. Altogether, we demonstrate that vRVFV-4s optimally combines efficacy with safety, holding great promise as a next-generation RVF vaccine.

## Introduction

Rift Valley fever virus (RVFV) is a mosquito-borne pathogen that is pathogenic to ruminants and humans. The virus, member of the *Phlebovirus* genus, family *Phenuiviridae*, order *Bunyavirales*^1^, causes abortions in pregnant ruminants and mortality among newborns. RVF outbreaks have devastating socio-economic consequences and the disease is notifiable to the World Organisation for Animal Health (OIE). In humans, the majority of RVFV infected individuals develop flu-like symptoms with fever, chills and malaise that usually resolve within 1–2 weeks. However, a small percentage of individuals develop encephalitis or haemorrhagic fever, the latter of which may have a fatal outcome^2,3^. About 10% of infected persons develop transient or permanent loss of vision resulting from retinal lesions, regardless of disease severity^3^.

Humans can become infected through contact with virus-contaminated animal products or via the bites of infected mosquitoes^2^, whereas transmission among ruminants strictly depends on mosquito vectors. The virus is currently confined to the African continent, several islands off the south-eastern African coast and the Arabian Peninsula, where it has been detected in more than 50 wild-caught mosquito species^4,5^. Vector competence experiments in laboratory settings have suggested that more than 65 mosquito species may be capable of transmitting the virus^5–9^. Globalization, climate change and the global prevalence of susceptible ruminants and mosquito vectors together call for awareness for outbreaks in yet unaffected territories and underscores the urgent need for appropriate, globally available control measures.

Vaccination is currently the only effective means of controlling RVF outbreaks. In several enzootic areas, inactivated- and live-attenuated vaccines are used to protect domesticated ruminants. Inactivated vaccines can be applied safely during all ruminant life stages, including very young and pregnant animals, although for optimal efficacy multiple administrations are required. In contrast, live-attenuated vaccines were shown to be effective in target species after a single vaccination^10–12^. The Smithburn strain was developed by attenuating a virulent strain by serial intracerebral passage in mice^13^. Vaccines based on this strain are very effective, although residual virulence prohibits use in very young and pregnant animals^14,15^. Another live-attenuated strain, named Clone 13, is a plaque-purified virus originating from a non-fatal human case^16^. Clone 13 was found to lack 69% of the NSs gene, encoding the major virulence factor of RVFV and was shown to be safe and effective in lambs^17^, cattle and pregnant ewes^18^. However, studies performed according to the European Pharmacopeia demonstrated that administration of a high dose results in vertical transmission and teratogenic effects^17^.

To optimally combine efficacy with safety, next-generation live-attenuated vaccines are being developed using reverse genetics. The RVFV reverse genetics system is based on three plasmids, each encoding one of the three viral genome segments in antigenomic-sense orientation: the large (L) segment encoding the viral polymerase, the small (S) segment encoding NSs and the nucleocapsid (N) protein and the M segment encoding a glycoprotein precursor, which is co-translationally cleaved into the structural glycoproteins Gn and Gc, a 14-kDa non-structural protein (NSm) and an accessory protein of 78-kDa, comprising the NSm and Gn coding regions. By means of reverse genetics, several recombinant viruses have been created with attenuating mutations and/or deletions, and have been evaluated in efficacy and safety trials with promising results.

Our laboratory previously developed a method of bunyavirus attenuation that is based on splitting the M genome segment. In this manner, the authentic open reading frame of the M segment, which encodes the two viral glycoproteins, Gn and Gc, was split into two fragments, each encoding for one of the two glycoproteins^19^. The resulting vaccine virus, which comprises four instead of three segments (RVFV-4s) and in addition lacks the major virulence factor NSs, was shown to be completely safe in even the most susceptible target species, young lambs and pregnant ewes.^28,29^ Apart from this strong safety profile, RVFV-4s was shown to provide protective immunity in lambs after a single immunization^29^.

In the present work, we further evaluated the safety and efficacy of the RVFV-4s vaccine for veterinary application (vRVFV-4s), according to the recommendations of the OIE Terrestrial Manual Chapter 2.1.18 and the Ph. Eur. 5.2.7 monograph. Our results show that vRVFV-4s does not disseminate in vaccinated animals and is not shed or spread to the environment. The virus does not cause viremia and does not revert to virulence, even after administration of a high dose in the most susceptible target species. Finally, a single vaccination was shown to provide protective immunity in lambs, goat kids and calves.

## Results

### vRVFV-4s does not disseminate in vaccinated animals and is not shed to the environment

**Fig. 1:**
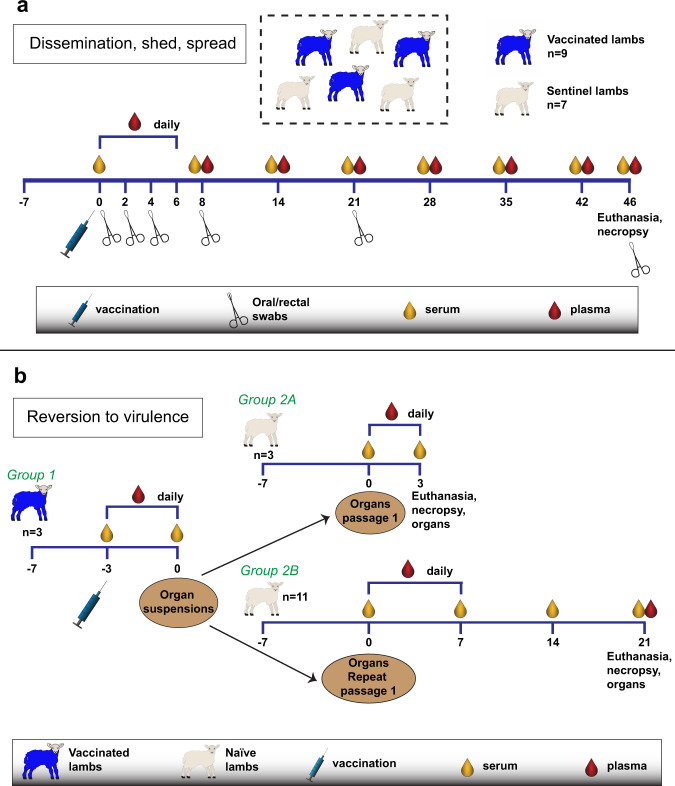
Schematic representation of the experimental setup of the safety trials. Experimenten setup of the (**a**) dissemination shedding and spreading study and the (**b**) reversion to virulence study with lambs.

Evaluation of the safety of live-attenuated vaccines according to the European pharmacopoeia requires demonstrating (1) lack of dissemination of the vaccine virus in vaccinated animals, (2) lack of shedding of the vaccine from vaccinated animals and (3) absence of vaccine spread from vaccinated to unvaccinated animals. To evaluate these safety requirements, a group of young lambs was vaccinated with a high dose (10^7.0^ TCID_50_) of vRVFV-4s and housed together with a group of sentinel lambs. Dissemination of the vaccine virus was assessed by determining vaccine viral RNA in daily collected plasma samples and in RVFV target organs (liver, spleen and lymph nodes) as well as testes collected at necropsy. Shedding was evaluated by assessing the presence of viral RNA in oral and rectal swabs and urine collected from vaccinated animals whereas spread was assessed by testing similar samples of the unvaccinated sentinel animals (Fig. 1a).

**Fig. 2:**
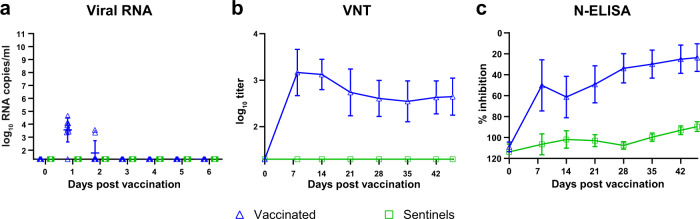
Viral RNA in plasma samples and serological responses in lambs of the DSS study. Monitoring of viremia (**a**) in inoculated and sentinel lambs by RT-qPCR. Samples that tested negative are depicted at the detection limit of the PCR (1.3 log_10_ RNA copies/ml). Neutralizing antibody responses as determined by VNT (**b**) in weekly obtained sera. Detection of anti-N antibodies by ELISA (**c**) in weekly obtained sera. Titres are expressed as percentage inhibition ratio of the optical densities (OD) of the sample and the OD of the negative control (% S/N). All values lower than 40% are considered positive, between 40 and 50% are considered doubtful and above 50% are considered negative. Measurements were taken from distinct samples. Error bars represent s.d.

No significant temperature increases or clinical signs were observed in either vaccinated or sentinel lambs. Low copy numbers of viral RNA were detected in plasma samples of vaccinated animals at 1 and 2 days post vaccination (DPV), whereas plasma samples collected 3 DPV were all negative (Fig. 2a). Importantly, all plasma samples of the sentinel animals and swab and organ samples of both vaccinated and sentinel animals were negative for viral RNA. Furthermore, all testis samples of vaccinated males (*n* = 7) and sentinel males (*n* = 3) were confirmed negative for viral RNA as well. Immunogenicity of the vaccine was confirmed by detection of RVFV neutralizing antibodies and anti-N antibodies (Fig. 2b, c). Neither RVFV neutralizing antibody titres nor anti-N antibodies were detected in sera from sentinel lambs. Altogether, these results show that vRVFV-4s does not disseminate, and is not shed or spread to the environment.

### vRVFV-4s does not revert to virulence upon animal passage

**Fig. 3:**
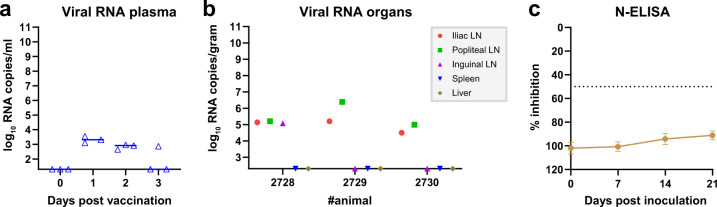
Viral RNA in plasma samples and serological responses in lambs of the RTV study. Monitoring of viremia (**a**) in lambs of Group 1 (vaccinated) by RT-qPCR. Samples that tested negative are depicted at the detection limit of the PCR (1.3 log_10_ RNA copies/ml). Results of Group 2 (first animal passage) are not depicted as no viral RNA was detected in plasma samples from these animals. Detection of viral RNA (**b**) in organ samples (3 days post vaccination) of lambs from Group 1. Results of Group 2 are not depicted as no viral RNA was detected in organ samples from Groups 2A (3 DPV) and 2B (21 DPV). LN lymph node. Detection of anti-N antibodies (**c**) by ELISA in weekly obtained sera of lambs from Group 2B. Titres are expressed as percentage inhibition ratio of the optical densities (OD) of the sample and the OD of the negative control (% S/N). All values lower than 40% are considered positive, between 40 and 50% are considered doubtful and above 50% are considered negative. Measurements were taken from distinct samples. Error bars represent s.d.

Another vaccine safety requirement is absence of reversion to virulence (RTV) upon animal passage. To address the possibility of RTV, three lambs were inoculated with vRVFV-4s and euthanized and necropsied at 3 DPV (Group 1) the moment of most active replication of wild-type RVFV (Fig. 1b). In line with the dissemination, shedding and spreading (DSS) study, low levels of viral RNA were detected in plasma samples collected the first DPV (Fig. 3a). No viral RNA was detected in liver and spleen samples, whereas in most of the lymph node samples, low levels of viral RNA were detected (Fig. 3b), with the exception of the inguinal lymph nodes of two lambs, which were negative. No infectious vaccine virus was recovered from any of the plasma or organ suspensions.

Because the popliteal lymph node suspensions contained the highest RNA copy numbers on average (Fig. 3b), the lymph node suspensions of lamb 2728, 2729 and 2730 were pooled and used to inoculate three naive lambs (Group 2A). These lambs were also euthanized at 3 days post inoculation. No clinical signs were observed and no viral RNA was detected in plasma and organ samples of Group 2A, suggesting that replication of vRVFV-4s in the initially vaccinated lambs was absent or very limited. To confirm these findings, another group of lambs (Group 2B; *n* = 11) was inoculated with the same lymph node suspension used to inoculate Group 2A. These lambs were monitored for 21 days. No clinical signs were noted during the observation period and no viral RNA was detected in any of the plasma samples or in the organ samples collected at necropsy. Furthermore, no antibodies were detected by N-ELISA in these animals (Fig. 3c). These findings confirm the inability to passage the vaccine virus from animal to animal and suggest that the chance of RTV of this vaccine in the field is negligible.

### vRVFV-4s efficacy in young ruminants following homologous challenge

**Fig. 4:**
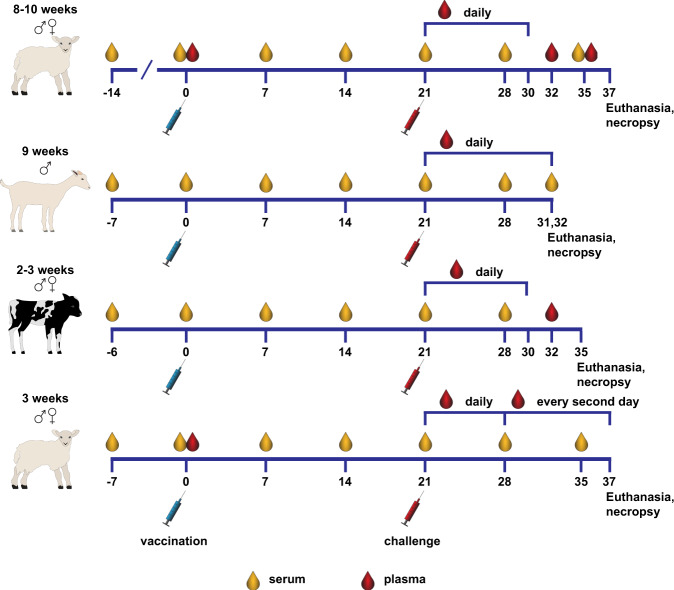
Schematic representation of the experimental setup of the efficacy trials. Experimental setup of a homologous vRVFV-4s vaccination-challenge trialwith lambs, goat kids and calves and a heterologous vRVFV-4s vaccination-challenge trial with lambs.

**Fig. 5:**
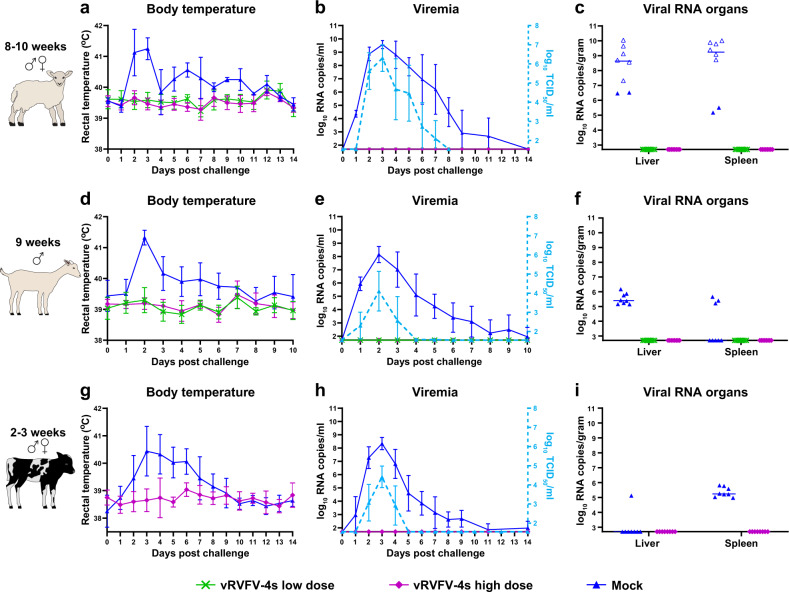
Body temperatures and viral RNA in plasma and organ samples measured during the efficacy trials. Average rectal temperatures of vRVFV-4s vaccinated and mock-vaccinated lambs, goat kids and calves (**a**, **d**, **g**) post homologous RVFV challenge. Monitoring of viremia in vaccinated and mock-vaccinated lambs by RT-qPCR and virus isolation (**b**, **e**, **h**). Samples that tested negative are depicted at the detection limit of the PCR (1.3 log_10_ RNA copies/ml) or virus isolation (1.55 log_10_ TCID_50_/ml). Detection of viral RNA in liver and spleen samples (**c**, **f**, **i**). The detection limit of the PCR was 2.3 log_10_ RNA copies/g. Open symbols represent animals that died or were euthanised during the study because they reached a humane endpoint. Measurements were taken from distinct samples.

**Fig. 6:**
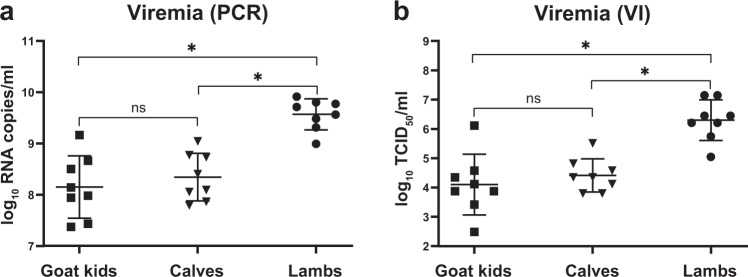
Comparison of peak viremia of mock-vaccinated goat kids, calves and lambs. Peak viremia, as detected by PCR (**a**) or virus isolation (**b**) of mock-vaccinated animals. For calves and lambs, values measured at 3 days post challenge are plotted, and for goat kids, values measured at 2 days post challenge. Each symbol depicts the value from an individual animal. Statistical significance of the differences between the groups was determined by one-way ANOVA with Tukey test for multiple comparison, (ns) not significant, asterisk (*) significant with a *p* value ≤ 0.05. Error bars represent s.d.

To investigate vaccine efficacy of vRVFV-4s in different ruminant species, the vaccine was applied to lambs, goat kids and calves (Fig. 4). Two vaccine doses were tested in lambs and goat kids, while in calves, only one dose was evaluated (Table 1). All animals were vaccinated once and challenged 21 days later with homologous RVFV, strain 35/74. After challenge, all control lambs developed fever and viremia (Fig. 5a, b). Six of the eight control lambs either died acutely or were euthanized when reaching a pre-defined humane endpoints (HEP). All mock-vaccinated calves and goat kids survived the RVFV challenge infection. Of note, one goat kid acutely succumbed at 6 DPI. Necropsy of this animal revealed a bacterial fibrinous pleuropneumonia. Viremia in the mock-vaccinated goat kids and calves was on average lower than in lambs (Fig. 6). In addition, virus could be isolated from plasma of both calves and goat kids only in a 3 day time span following challenge, as compared with 6 days in lambs (Fig. 5b, e, h). High levels of viral RNA were detected in liver and spleen samples derived from lambs that either died or were euthanized when a HEP was reached (Fig. 5c). Low levels of viral RNA were detected in liver and spleen samples of surviving mock-vaccinated lambs (Fig. 5c), goat kids (Fig. 5f) and calves (Fig. 5i). Of note, viral RNA was detectable in the livers of all mock-vaccinated goat kids and in spleens of three animals whereas only one mock-vaccinated calf had detectable levels of viral RNA in the liver, while all mock-vaccinated calves contained viral RNA in the spleen at necropsy.

**Table 1:** Experimental details animal trials.

Trial	DSS	RTV	Efficacy sheep	Efficacy goats	Efficacy cattle	HTC
Animal species	Sheep	Sheep	Sheep	Goats	Cattle	Sheep
Breed	Texel cross	Texel cross	Texel cross	Saanen	Holstein-Friesian	Texel cross
Age (weeks)	3	3	8–10	9	2–3	3
Gender	Males and females	Males and females	Males and females	Males	Males and females	Males and females
No. per group	9, 7	3, 3, 11	8	8	8	10
Groups	Inoculated Sentinels	1A 2A 2B	Low dose High dose Mock	Low dose High dose Mock	Vaccine Mock	Vaccine Mock
Vaccine batch	MSV	MSV	MSV+5	MSV+2	MSV+2	MSV+5
Vaccine dose (TCID_50_)	10^7^	10^7^	10^4.5^ 10^5.5^ –	10^5^ 10^6^ –	10^6^ –	10^5.5^ –
Challenge dose (TCID_50_)	–	–	10^5^	10^5^	10^5^	10^5^
Challenge strain			rRVFV 35/74	rRVFV 35/74	rRVFV 35/74	RVFV-ZH501

**Fig. 7:**
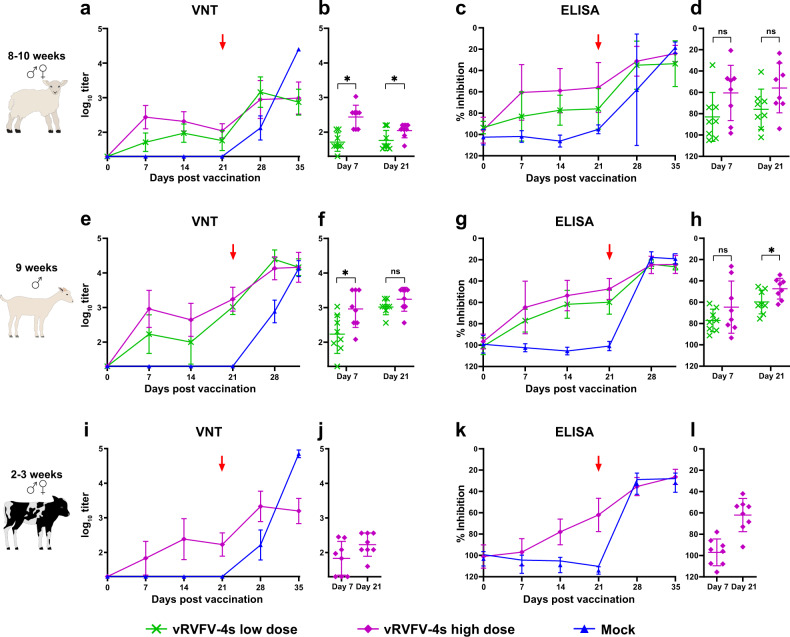
Serology results of the vRVFV-4s efficacy trials. Neutralizing antibody responses in time as determined by VNT (**a**, **e**, **i**). Challenge (red arrow) of both groups was on DPV 21. Statistical analysis by unpaired *T*-test of the VNT responses 1 week post vaccination and at the day of challenge (**b**, **f**, **j**). Detection of anti-N antibodies by ELISA in weekly obtained sera (**c**, **g**, **k**). Titres are expressed as percentage competition ratio of the optical densities (OD) of the sample and the OD of the negative control (% S/N). All values below 40% are considered positive, between 40 and 50% are considered doubtful and above 50% are considered negative. Statistical analysis by unpaired *T*-test of the N-ELISA responses 1 week post vaccination and at the day of challenge (**d**, **h**, **l**), (ns) not significant, asterisk (*) significant with a *p* value ≤ 0.05. Measurements were taken from distinct samples. Error bars represent s.d.

**Fig. 8:**
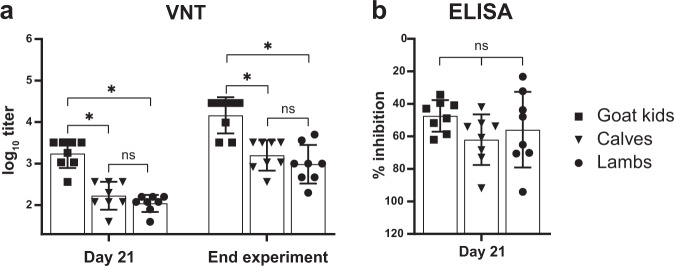
Interspecies comparison of the antibody response elicited by vRVFV-4s. Only responses elicited by the high vaccine dose are shown. Neutralizing titres (**a**) and anti-N-ELISA titres (**b**), measured at the day of challenge and at the day of termination, are plotted as individual values with averages and s.d. Statistical significance of the differences between the groups was determined by one-way ANOVA with Tukey test for multiple comparison, (ns) not significant, asterisk (*) significant with a *p* value ≤ 0.05. Measurements were taken from distinct samples.

In contrast to the mock-vaccinated animals, none of the vRVFV-4s-vaccinated animals presented with an elevated body temperature. Also no viral RNA was detected in any of the plasma, liver and spleen samples. This complete protection is probably attributed to the robust neutralizing antibody responses induced by vaccination with vRVFV-4s, as determined by VNT. All animals, regardless of vaccine dose or species, seroconverted by day 21 post vaccination (Fig. 7). Of note, following challenge, a boost in both neutralizing and anti-N antibody titres were observed in all vaccination groups. In general, a dose effect was observed in both lambs and goat kids in which a higher dose resulted in a more rapid and higher overall response. Clear differences were also observed between species (Fig. 8). Significantly higher neutralizing antibody responses were measured in goat kids, as compared with lambs and calves, both on the day of challenge and at the end of the in-life phase (Fig. 8). Contrary, lambs and calves had comparable neutralizing titres at both time points. Overall, these studies showed that vRVFV-4s efficiently protects multiple ruminant species from homologous RVFV challenge with a single vaccination.

### vRVFV-4s efficacy in young lambs following heterologous challenge

After demonstrating full protection against a homologous challenge in the lamb, goat and cattle model, we investigated whether vRVFV-4s also provides immunity against a genetically distinct RVFV isolate (ZH501). Previous vRVFV-4s vaccine efficacy trials were performed with lambs between 8 and 14 weeks of age. To evaluate efficacy in the even younger lambs, the present study was performed with lambs 3–4 weeks old at the moment of vaccination. Experiments with lambs of 3–4 weeks old is challenging, as the immune system of lambs of this age is not developed completely. Therefore, to anticipate on some loss of lambs due to unforeseen events, ten lambs per group were enroled in this experiment. Of note, to refine the experiment and prevent unnecessary discomfort, all mock-vaccinated animals were euthanized at day 4 after challenge.

**Fig. 9:**
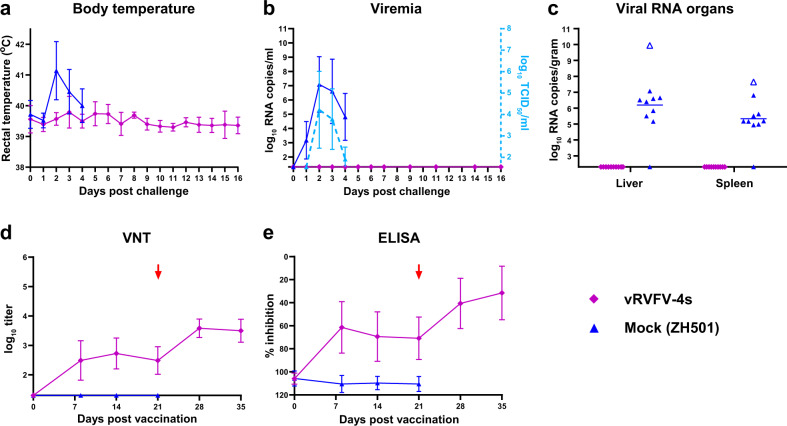
Results heterologous challenge trial. Average rectal temperatures (mean) with s.d. of vRVFV-4s vaccinated and mock-vaccinated lambs post heterologous RVFV challenge (**a**). Monitoring of viremia in vaccinated and mock-vaccinated lambs by RT-qPCR and virus isolation (**b**). Samples that tested negative are depicted at the detection limit of the PCR (1.3 log_10_ RNA copies/ml) or virus isolation (1.55 log_10_ TCID_50_/ml). Detection of viral RNA in liver and spleen samples (**c**). The detection limit of the PCR was 2.3 log_10_ RNA copies/g. Open symbols represent animals that died or were euthanised during the study because they reached a humane endpoint. Neutralizing antibody responses in time as determined by virus-neutralizing test (**d**). Challenge (red arrow) of both groups was on study day 21. Detection of anti-N antibodies by ELISA (**e**) in weekly obtained sera. Titres are expressed as percentage competition ratio of the optical densities (OD) of the sample and the OD of the negative control (% S/N). All values lower than 40% are considered positive, between 40 and 50% are considered doubtful and above 50% are considered negative. Challenge (red arrow) of both groups was on DPV 21. Measurements were taken from distinct samples.

In line with our previous challenge experiments with strain 35/74, all mock-vaccinated lambs developed viremia associated with elevated body temperature (Fig. 9a, b). Compared with strain 35/74, viremia of strain ZH501 was on average lower and a higher variation was observed (compare Fig. 5b and Fig. 9b). One animal died acutely at day 2 post infection, and presented with high viral loads in liver and spleen compared with the rest of the mock-vaccinated animals, which were euthanized at day 4 post infection (Fig. 9c). Although all control lambs developed viremia, one animal was negative for viral RNA in liver and spleen at the time of necropsy. Importantly, none of the vRVFV-4s-vaccinated lambs developed fever or viremia and all organ samples of the vaccinated animals were negative for viral RNA at the end of the experiment. Antibody responses elicited by vRVFV-4s in this lamb experiment resembled closely those in older lambs, described above, although variation of the neutralizing titres between animals was larger (compare Fig. 7a, c and Fig. 9d, e). Mock-vaccinated animals remained sero-negative until necropsy at day 4 post challenge.

## Discussion

The present work demonstrates that vRVFV-4s can be applied safely in the most susceptible target species and that the vaccine does not spread to the environment. Application of an overdose of vRVFV-4s in young lambs revealed absence of viremia and absence of dissemination to target organs, absence of shedding to the environment and absence of spreading to in-contact animals. We also demonstrate that vRVFV-4s cannot be recovered from vaccinated animals, suggesting that very limited or absent replication renders the risk of RTV negligible. The present study also expands previous vaccine efficacy studies, by demonstrating that a single vaccination induces protective immunity in the three major target species of RVFV: lambs, goat kids and calves. Finally, we demonstrate that a single vaccination of vRVFV-4s provides protective immunity against a genetically and geographically distinct strain of RVFV.

Although the vRVFV-4s vaccine induced protective immunity in all animals, notable differences between species were observed in mock-vaccinated control groups. In lambs, viremia was higher and of longer duration as compared with viremia in goat kids and calves. These observations are in line with observations from others^20^ and underscore the notion that lambs play a dominant role in the transmission of RVFV via mosquitoes. Differences between species were also noted when analysing neutralizing antibody responses. The most striking observation was that neutralizing antibody responses were significantly higher in goat kids than in lambs and calves. Similar observations have been reported after vaccination with Clone 13^12^ and after vaccination with a chimpanzee adenovirus-vectored RVF vaccine (ChAdOx1-GnGc)^20^, suggesting that it is an intrinsic feature of goats to mount a superior antibody response to vaccines, although this may not be related to an overall better protective immunity. Another interesting observation is that VNT titres, in contrast to the N-ELISA values, temporarily declined for 1–2 weeks, depending on the species, followed by a gradual increase. This is possibly explained by the class-switch from IgM to IgG antibodies and a difference in neutralizing activity of IgM and IgG antibodies and calls for further research into the contribution of different antibody isotypes, antibody subclasses and affinity maturation to neutralization of RVFV.

Results of previous studies in which efficacy of live-attenuated RVF vaccines in cattle was addressed are variable. Vaccination of small groups of cattle with Clone 13 or the Smithburn vaccine resulted in neutralizing antibody responses in all vaccinated animals^10^. However, another small-group study reported that a single and even a booster vaccination with the Smithburn vaccine resulted in lower antibody responses then vaccination with an inactivated vaccine^21^. In studies with larger test groups, vaccination with neither the Smithburn strain nor Clone 13 resulted in seroconversion in all vaccinated cattle^12,15^. Due to the possible lower immunogenicity of live-attenuated RVF vaccines in cattle as compared with sheep and goats, we applied a higher dose (10^6^ TCID_50_/ml) in this species. Considering that this vaccination resulted in protective immunity, additional studies are required to assess whether calves can also be protected with a lower dose of vRVFV-4s.

Recent phylogenetic analyses of RVFV isolates have grouped the virus into 7 or 15 lineages, depending on the number of strains included^22,23^, with reported maximum pairwise differences between distant strains of 5.4% at the nucleotide level and 2.8% at the amino acid level. Nevertheless, neutralizing antibodies against RVFV are believed to be active against all circulating strains as RVFV seems to comprise only a single serotype. Underscoring this notion, vaccination of young lambs with vRVFV-4s provided protective immunity against RVFV strain ZH501, a genetically and geographically distinct strain.

In conclusion, the studies reported here confirm the high safety profile of vRVFV-4s and demonstrate that a single vaccination provides protective immunity in all three major target species of RVFV.

## Methods

### Ethics statement

Animal testing and research was in compliance with all relevant ethical regulations. Animal trials were conducted in accordance with European regulations (EU directive 2010/63/EU) and the Dutch Law on Animal Experiments (Wod, ID number BWBR0003081). Permissions were granted by the Dutch Central Authority for Scientific Procedures on Animals (Permit Numbers: AVD401002017816 and AVD4010020187168). Specific procedures were approved by the Animal Ethics Committees of Wageningen Research. The following HEP were applied: (1) the animal is recumbent and does not rise even after stimulation, (2) the animal is unable to drink and (3) the animal is lethargic (listless, apathic, non-responsive to stimuli).

### Animals

All animals used in the animal trials were purchased from conventional Dutch farms and were clinically healthy, as assessed by a veterinarian, before and upon arrival at the animal facilities (Table 1). The animals were distributed randomly over groups and housed in a BSL-3 containment facility. Animals were allowed to acclimatize for 7 days. In case animals were enroled in a study below 1 month of age, animals received bovine colostrum and artificial milk directly from birth. To ensure HEP were recognized timely, animals were clinically assessed daily and during critical periods, twice or three times per day. Rectal temperatures were recorded daily.

### Cells and viruses

The RVFV-4s vaccine virus used in the presented studies is based on RVFV strain 35/74^24^ and referred to as vRVFV-4s. A master seed virus (MSV) of vRVFV-4s was prepared by amplification of the virus in BSR-T7 cells, grown in GMEM medium (Gibco), supplemented with 5% foetal calf serum (FCS, SAFC), 4% tryptose phosphate broth (Gibco), 1% non-essential amino acids (Gibco) and 0.01% Gentamicin. The MSV was passed 2–5 times in BSR-T7 cells and was subsequently used for efficacy studies with goats, calves and lambs (Table 1). Identity of the vRVFV-4s virus batches was confirmed by conventional RT-PCR and next-generation sequencing. For homologous challenge, recombinant RVFV strain 35/74 (rRVFV 35/74) was used and for heterologous challenge wild-type RVFV strain ZH501 was used. rRVFV 35/74 was propagated in BHK-21 cells, which were grown in CO_2_-independent medium (Gibco), supplemented with 5% FCS (Gibco), 1% l-glutamine (Gibco) and 1% antibiotic/antimycotic (anti/anti, Gibco). RVFV strain ZH501 was propagated in Vero E6 cells that were grown in EMEM (Gibco), supplemented with 5% FCS (Gibco) and 1% anti/anti. For the virus neutralization assay, BHK-21 cells were used, which were grown in GMEM medium, supplemented with 5% FCS, 1% l-glutamine and 1% anti/anti.

### DSS study

In the DSS study, nine 4-week-old lambs were inoculated intramuscularly (IM), in the right thigh muscle (*biceps femoris*) with 10^7.0^ TCID_50_ (in 1 ml) of vRVFV-4s MSV and housed together with seven unvaccinated (sentinel) lambs. Serum, plasma, oral and rectal swab samples were collected from all animals as indicated (Fig. 1a). Four hours post vaccination temperatures were measured to assess acute reactions to vaccination. At the end of the observation period, on day 46 post inoculation, animals were euthanized and samples from spleen, liver, lymph nodes, testes and urine were obtained.

### RTV study

In phase 1 of the RTV study, three 4-week-old lambs (Group 1) were inoculated IM (*biceps femoris*) with 10^7.0^ TCID_50_ (in 1 ml) of vRVFV-4s MSV (Fig. 1b). Three days post inoculation, a full necropsy was performed. Presence of viral RNA in plasma, liver, spleen and lymph node suspensions was determined by quantitative reverse transcriptase polymerase chain reaction (RT-qPCR) and the samples with the highest viral RNA loads were pooled and used to inoculate another group of three 4-week-old lambs, also via IM administration (*biceps femoris*, 1 ml) (Group 2A). Similar samples were collected from this group, which were euthanized at 3 days post inoculation. Subsequently in phase 2 of the study, the same organ suspension pool from the first group of vaccinated lambs (Group 1) was used as inoculum (also applied IM) for a larger group of 4-week-old lambs (*n* = 12), Group 2B. Lambs of Group 2B were monitored for 3 weeks following inoculation and plasma, serum and organ samples were collected at the time points as indicated (Fig. 1b).

### Efficacy studies

All vaccinations were applied IM (*biceps femoris*) and all challenges intravenously. For mock vaccinations (control group) and vaccine dilutions 0.9% NaCl was used. Details of the individual efficacy trials are summarized in Table 1 and Fig. 4.

### Preparation of organ suspensions

Ten percent organ homogenates were prepared using the ULTRA-TURRAX system in combination with DT-20 tubes (IKA, Staufen, Germany). Briefly, 0.6 g tissue was homogenized in 6 ml culture medium for 40 s followed by removal of cell debris by slow-speed centrifugation. The suspensions were used for virus detection by RT-qPCR and virus isolation.

### RNA isolation and RT-qPCR

Viral RNA was isolated with the NucliSENS easyMAG system according the manufacturer’s instructions (bioMerieux, France) from either 0.5 ml of plasma or 0.5 ml of 10% organ suspension. Briefly, 5 µl RNA was used in a RVFV RT-qPCR using the LightCycler one-tube RNA Amplification Kit HybProbe (Roche, Almere, The Netherlands) in combination with a LightCycler 480 real-time PCR system (Roche) and the RVS forward primers (AAAGGAACAATGGACTCTGGTCA), the RVAs (CACTTCTTACTACCATGTCCTCCAAT) reverse primer and a FAM-labelled probe RVP (AAAGCTTTGATATCTCTCAGTGCCCCAA)^25^. Virus isolation was performed on RT-qPCR positive samples with a threshold above 10^5^ RNA copies/ml as this has been previously shown to be a cut-off point below which no live virus can be isolated.

### Virus isolation

Plasma or 10% organ suspensions were used for virus isolations. BHK-21 cells were seeded at a density of 20,000 cells/well in 96-well plates. Serial dilutions of samples were incubated with the cells for 1.5 h before medium replacement. Cytopathic effect was evaluated after 5–7 days post infection and tissue culture infective dose 50 (TCID_50_) was calculated using the Spearman–Kärber algorithm^26,27^.

### Serology

A virus neutralization test was performed using a RVFV-4s variant encoding GFP (RVFV-4s_GFP_)^19,28^. Briefly, twofold serial dilutions of inactivated (2 h at 56 °C) sera were mixed with a fixed amount of virus (~200 TCID_50_) in 96-well plates. After a 2 h incubation period, 20,000 BHK-21 cells were added to each well and plates were incubated for 2 days at 37 °C and 5% CO_2_, followed by evaluation of GFP expression. Neutralizing titres were determined using the Spearman–Kärber algorithm.

A commercial competitive ELISA (RIFTC, ID-VET Montpellier, France) was used to detect antibodies against the RVFV nucleoprotein (anti-N antibodies), following the manufacturer’s instructions. The method measures percentage competition between antibodies present in test sera and a monoclonal antibody.

### Statistical analysis

Statistical significance was determined by one-way ANOVA and Tukey test for multiple comparison and *t*-tests, using GraphPad Prism 8. The significant threshold was set to *p* values ≤ 0.05.

### Reporting summary

Further information on research design is available in the Nature Research Reporting Summary linked to this article.

## Supplementary information

Reporting Summary

## Data Availability

All data generated or analyzed during this study are included in this published article.
